# Exploration of Antibiotic Activity of Aminoglycosides, in Particular Ribostamycin Alone and in Combination With Ethylenediaminetetraacetic Acid Against Pathogenic Bacteria

**DOI:** 10.3389/fmicb.2020.01718

**Published:** 2020-07-29

**Authors:** Jing Kong, Zhuo-Xun Wu, Liuya Wei, Zhe-Sheng Chen, Sabesan Yoganathan

**Affiliations:** ^1^Department of Pharmaceutical Sciences, College of Pharmacy and Health Sciences, St. John’s University, New York, NY, United States; ^2^School of Pharmacy, Weifang Medical University, Weifang, China

**Keywords:** antibiotic, aminoglycoside, drug resistance, *Escherichia coli*, ethylenediaminetetraacetic acid, infection, netilmicin, ribostamycin

## Abstract

The emergence of infections caused by bacterial pathogens that are resistant to current antibiotic therapy is a critical healthcare challenge. Aminoglycosides are natural antibiotics with broad spectrum of activity; however, their clinical use is limited due to considerable nephrotoxicity. Moreover, drug-resistant bacteria that cause infections in human as well as livestock are less responsive to conventional antibiotics. Herein, we report the *in vitro* antibacterial evaluation of five different aminoglycosides, including ribostamycin, against a panel of Gram-positive and Gram-negative pathogens. Eight of the tested bacterial strains are linked to gastrointestinal (GI) infections. The minimum inhibitory concentration (MIC) of ribostamycin against three different *Escherichia coli* strains is in the range of 0.9–7.2 μM and against a strain of *Haemophilus influenzae* is 0.5 μM. We also found that the MIC of ribostamycin was considerably enhanced from 57.2 to 7.2 μM, an 8-fold improvement, when bacteria were treated with a combination of ribostamycin and ethylenediaminetetraacetic acid (EDTA). These findings demonstrate a promising approach to enhance the clinical potential of ribostamycin and provide a rational for its antibiotic reclassification from special level to non-restricted level.

## Introduction

Infections caused by emerging pathogenic bacteria, including those that are resistant to commonly used antibiotics, have become a major global health problem (Parks et al., 2012; Beceiro et al., 2013). The Centers for Disease Control and Prevention (CDC) estimates that more than 2.8 million infections in the United States are caused by antibiotic-resistant bacteria. Moreover, approximately 35,000 people die due to infections caused by drug-resistant bacteria (CDC, 2019). Infections caused by drug-resistant bacterial pathogens have become a major clinical threat in the recent years (Woodford et al., 2011; Yoganathan et al., 2013; Stryjewski and Corey, 2014; Yoganathan and Miller, 2015; Xu et al., 2016; Frieri et al., 2017). More specifically, the outer membrane in Gram-negative bacteria is an additional barrier to overcome when treating infections caused by such pathogens (Nikaido and Nakae, 1980; Yoganathan et al., 2011; Steinbuch and Fridman, 2016; Masi et al., 2017; Wright et al., 2017). Many of the Gram-positive and Gram-negative pathogens are also the cause of infection in livestock, leading to challenges in veterinary medicine (Hillerton and Berry, 2005; Schabauer et al., 2018). Hence, there is a pressing need to develop new antibiotic leads and new methods to sensitize existing antibiotics against Gram-negative bacteria.

**Figure 1:**
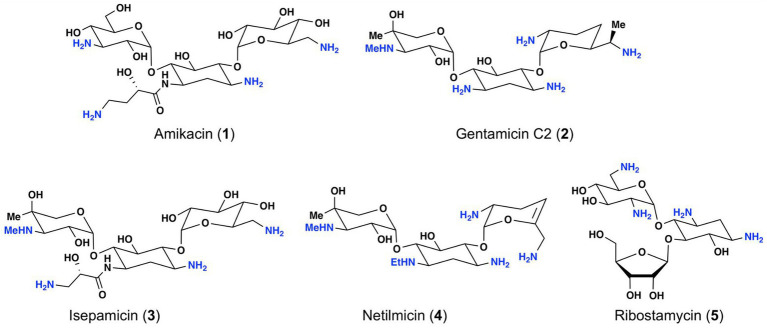
Structures of selected aminoglycoside antibiotics.

Among the different classes of antibiotics currently available for treatment of bacterial infections, aminoglycosides represent a structurally unique natural antibiotic class (Houghton et al., 2010; Woodford et al., 2011; Krause et al., 2016). Due to the presence of several amino groups, aminoglycosides are characterized as cationic molecules (Figure 1). Some of the common aminoglycosides that are either clinically used or exhibit clinical potential include gentamicin, amikacin, netilmicin, isepamicin, and ribostamycin (Figure 1; Durante-Mangoni et al., 2009; Avent et al., 2011).

Aminoglycosides are potent and broad-spectrum antibiotics. Their primary mechanism of action relates to inhibition of bacterial protein synthesis *via* binding to bacterial 30S ribosomal subunit through hydrogen bond and ionic interactions (Kotra et al., 2000; Magnet and Blanchard, 2005; Kapoor et al., 2017). The positive charges of aminoglycosides have also been known to disrupt the integrity of bacterial cell membrane, which leads to pore-formation and subsequent cell death (Kotra et al., 2000; Magnet and Blanchard, 2005). Gentamicin is one of the aminoglycosides widely used in combination with other aminoglycosides for treating bacterial infections caused by *Pseudomonas aeruginosa*, *Staphylococcus aureus*, *Enterobacter*, and other pathogens (Hancock, 1981). Amikacin is another potent aminoglycoside, which is usually used for the treatment of aminoglycoside resistant infections (Vaara, 1992; Krause et al., 2016; Ramirez and Tolmasky, 2017). Netilmicin is a semisynthetic aminoglycoside, which shows similar activity as gentamicin with less toxicity (Miller et al., 1976). Isepamicin exhibits similar antibiotic spectrum as amikacin and is active against various Gram-negative bacteria (Falagas et al., 2012). Ribostamycin is a natural aminoglycoside, which has a broad-spectrum antibiotic activity (Horibe et al., 2001). Despite their broad-spectrum activity, nephrotoxicity and ototoxicity of aminoglycosides have been major adverse effects that limit their clinical use. The cationic property associated with the amino groups within this class of antibiotics is thought to play an important role in their toxicity profile (Lopez-Novoa et al., 2011). Typically, aminoglycoside associated adverse effects are managed *via* an extended interval dosing regimen, monitoring serum concentration following administration and avoiding co-administration of diuretics, nephrotoxic agents, or ototoxic medicines (Tulkens, 1999; Nakashima et al., 2000; Beauchamp and Labrecque, 2001; Rizzi and Hirose, 2007; Selimoglu, 2007). Bacteria develop resistance following prolonged usage of aminoglycosides in clinical settings (Jana and Deb, 2006; Vong and Auclair, 2012; Garneau-Tsodikova and Labby, 2016). Since the utility of clinically available aminoglycosides is greatly limited due to adverse toxicity and drug-resistance, it is important to identify new members of this class of antibiotics that exhibit low toxicity and improved efficacy against pathogenic bacteria.

Within the given members of aminoglycosides in Figure 1, ribostamycin is not on the list for routine clinical use in North America, Europe, or China (Avent et al., 2011 and Krause et al., 2016). Additionally, it is claimed to be classified as a “special level” antibiotic in China due to limited data available on the antibiotic spectrum and toxicity. Similarly, netilmicin and isepamicin, which are also not used clinically worldwide, are also classified as “restricted level” antibiotics in China. In an attempt to better understand the antibiotic spectrum and nephrotoxicity of these less popular aminoglycosides, we evaluated their antibiotic activity against a panel of Gram-positive and Gram-negative bacteria. To the best of our understanding, ribostamycin has not been tested against many clinically relevant bacterial strains, and there has not been any attempt to improve its efficacy against bacteria that are resistant to ribostamycin. The purpose of this work is to disclose the bioactivity data for ribostamycin and investigate its toxicity profile compared to gentamicin. The data may help researchers to consider ribostamycin as a non-restricted level antibiotic for clinical studies. Herein, we disclose the antibacterial and nephrotoxicity evaluation of ribostamycin, along with gentamicin, amikacin, netilmicin, and isepamicin. We also report the utility of ethylenediaminetetraacetic acid (EDTA) as an adjuvant to improve the efficacy of ribostamycin against Gram-negative bacteria.

## Materials and Methods

### Reagents

Dulbecco’s Modified Eagle’s Medium (DMEM), fetal bovine serum (FBS), 0.25% trypsin, and penicillin/streptomycin were purchased from Corning Incorporated (Corning, NY). 3-(4,5-dimethylthiazol-2-yl)-2,5-diphenyl tetrazolium bromide (MTT) powder and dimethylsulfoxide (DMSO) were bought from Sigma-Aldrich (St. Louis, MO). Antibiotics were purchased from the following vendors: gentamicin sulfate (VWR®), amikacin (MP Biomedicals), netilmicin sulfate (AK Scientific), and isepamicin sulfate (AK Scientific). Ethylenediaminetetraacetic acid tetrasodium salt, resazurin sodium salt, and lysogeny broth (LB) media were purchased from VWR.

### Cell Lines and Cell Culture

Human embryonic kidney (HEK293) cells and Madin-Darby Canine Kidney (MDCKII) cells were selected to perform the nephrotoxicity assay. Each cell line was cultured in DMEM medium containing 10% FBS and 1% penicillin/streptomycin at 37°C in a humidified incubator containing 5% CO_2_. HEK293 and MDCKII cells were grown as an adherent monolayer. Target bacterial strains, *Escherichia coli* (ATCC 25922, ATCC 4157, ATCC 12435, and ATCC 10798), *P. aeruginosa* (ATCC 27853), *Haemophilus influenzae* (ATCC 49247), *Staphylococcus epidermidis* (ATCC 12228), *Sta. aureus* (ATCC 12600), *Enterococcus faecalis* (ATCC 29212), and *Streptococcus pneumoniae* (ATCC 49619), were purchased from ATCC. All bacterial strains were grown in LB medium at 37°C in an incubator, with shaking at 200 rpm.

### Minimum Inhibitory Concentration Assay

#### MIC Determination

Bacteria were first inoculated in 10 ml of LB for 16 h in shaker at 37°C. The next day, 100 μl of the inoculum was transferred to a test tube with fresh media to obtain an inoculum with ~5 × 10^5^ CFU/ml (EUCAST of ESCMID, 2003). All aminoglycosides were dissolved in water to prepare samples for testing. Drugs were prepared as serially diluted concentration of 64, 32, 16, 8, 4, 2, 1, 0.5, and 0.25 μg/ml and 100 μl was transferred into a 96-well plate (Corning #353072). Then, 100 μl of 5 × 10^5^ CFU/ml inoculum was transferred into each well with the test drug. The plate was then incubated at 37°C for 14 h. The OD_600_ was recorded using microplate reader (ELx808). MICs are reported as the lowest concentration at which no bacterial growth was observed.

#### MIC Determination for Combination With EDTA

Ribostamycin solution (100 μl) with different concentrations (64, 32, 16, 8, 4, 2, 1, and 0.5–0.25 μg/ml) was added to each well. Then, 5 μl of EDTA (1 mg/ml) solution was added to each well. Subsequently, 100 μl of ~5 × 10^5^ CFU/ml inoculum was added into each well. The plates were then incubated at 37°C for 14 h. The OD_600_ was recorded using a microplate reader. MICs are reported as the lowest concentration at which no bacterial growth was observed.

#### MIC Determination Using Resazurin Dye

Sterile resazurin solution (0.02% by weight in water) was added into the 96-well plate after 16 h of incubation in a plate reader. The plate was further incubated for another 3 h to let the bacteria react with the resazurin dye at 37°C. After incubation, color change was used to estimate the MIC values. The well color changed from blue to purple/pink was estimated to be the MIC value.

#### Cytotoxicity Assay

HEK293 and MDCKII cells were selected to perform the nephrotoxicity assay. Nephrotoxicity of the antibiotics was determined by the MTT assay (Carmichael et al., 1987). Briefly, cells were collected and seeded evenly into 96-well plates (5 × 10^3^ cells per well) and were maintained overnight. At the next day, different concentrations of each antibiotics were added into the designated wells. After 68 h of incubation, 20 μl MTT solution (4 mg/ml) was added to each well and the cells were further incubated for additional 4 h in the incubator. Then, the supernatant was discarded and 100 μl of DMSO was added to dissolve the formazan crystals. The light absorbance was determined by using an accuSkan™ GO UV/Vis Microplate Spectrophotometer (Fisher Scientific, Fair Lawn, NJ) at a wavelength of 570 nm.

## Results

### Bacterial Susceptibility to Aminoglycosides

The *in vitro* activity of five different aminoglycosides (gentamicin, amikacin, netilmicin, isepamicin, and ribostamycin) was evaluated against a panel of Gram-negative and Gram-positive bacteria: *Es. coli* (ATCC 25922), *P. aeruginosa* (ATCC 27853), *H. influenzae* (ATCC 49247), *Sta. epidermidis* (ATCC 12228), *Sta. aureus* (ATCC 12600), *En. faecalis* (ATCC 29212), and *Str. pneumoniae* (ATCC 49619). The bacterial strains selected for this study are linked to various human infections, including respiratory, urinary tract (UT), bacteremia, or gastrointestinal (GI) infections. *En. faecalis* is typically characterized as a gut commensal organism; however, it is linked to frequent and serious infections, including UT infection (UTI) and bacteremia (Shankar et al., 2001). *Sta. aureus* is also part of commensal flora, yet a major cause of human infections, including skin, soft-tissue, respiratory, and bacteremia. Moreover, it has been reported that *Sta. aureus* is linked to UTI in hospitalized patients with intravenous catheter-related phlebitis and patients in long-term care facilities (Muder et al., 2006; Baraboutis et al., 2010).

Minimum inhibitory concentration values of aminoglycosides were determined using a microplate assay, where test samples were added to each well containing the bacterial inoculum (~5 × 10^5^ CFU/ml). All antibiotics were dissolved in water to prepare the stock solutions. The effectiveness of the compounds was measured over a 14-h period by measuring the optical density at 600 nm using a BioTek ELx808 Microplate Reader (EUCAST, 2003; Clinical and Laboratory Standards Institute, 2012).

**Figure 2:**
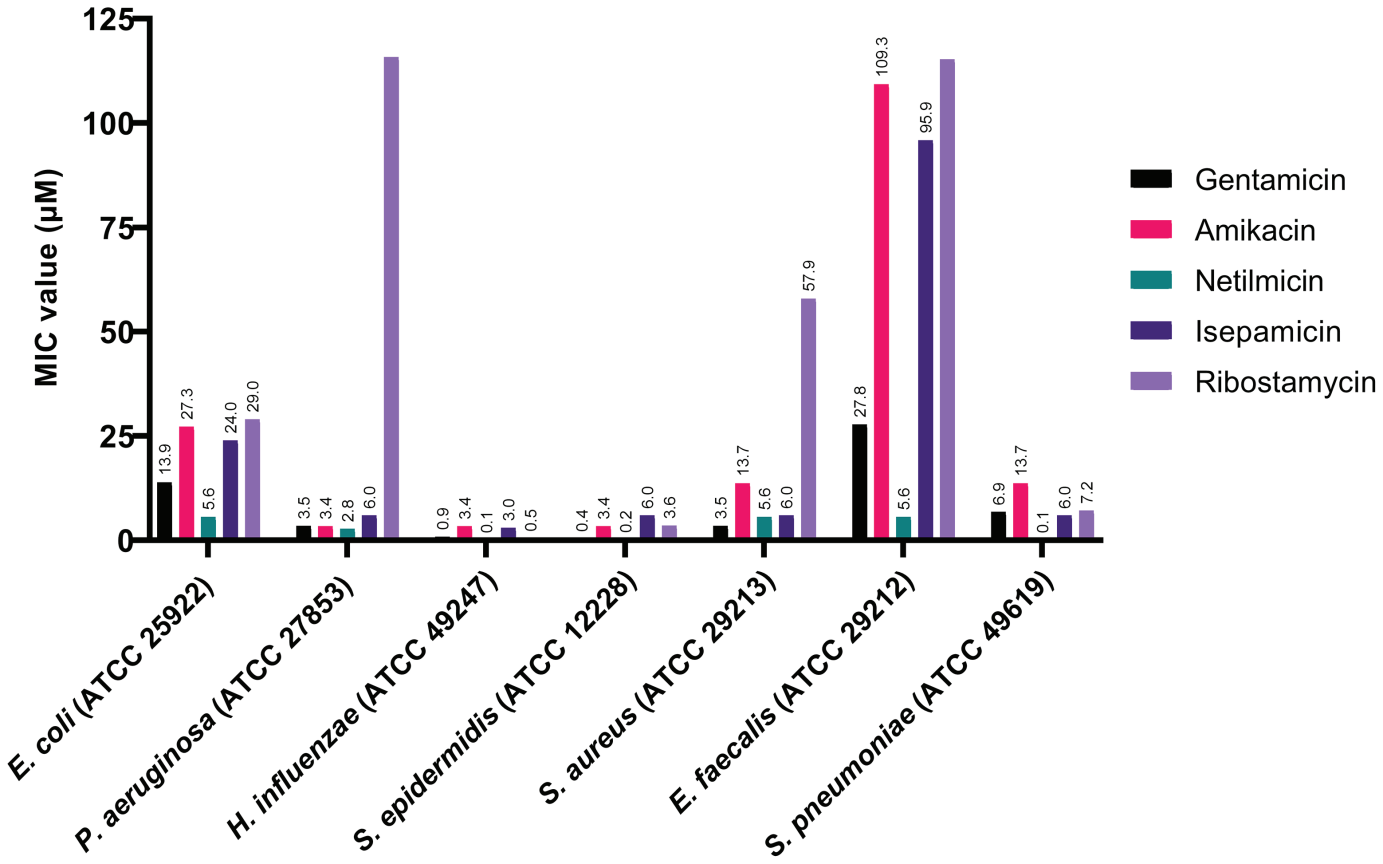
Antibacterial activity of aminoglycosides against selected Gram-positive and Gram-negative bacteria.

As an initial screening, we tested these five aminoglycosides against seven representative bacteria. The MIC values from the evaluation are given in Figure 2. We observed that all amino-glycosides are very potent against *H. influenzae*, *Sta. epidermidis*, and *Str. pneumoniae*, with MIC values between 0.1 and 13.7 μM. All seven bacterial strains tested are sensitive to netilmicin, with MIC values between 0.1–5.6 μM. The MIC value of ribostamycin against *Es. coli* was moderate (MIC = 29.0 μM) and very weak against *P. aeruginosa*, *Sta. aureus*, and *En. faecalis* (MIC > 115.8 μM). However, ribostamycin showed very potent MIC of 0.5 and 7.2 μM against *H. influenzae* and *Str. pneumoniae*, respectively.

**Figure 3:**
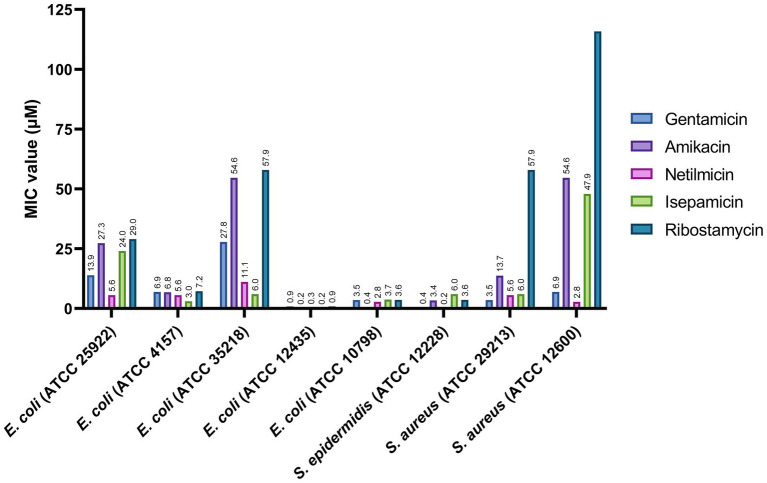
Antibacterial activity of aminoglycosides against selected *Escherichia coli* and *Staphylococcus* pathogens.

As a next phase, we investigated the potential of ribostamycin as a new antibiotic for the treatment of infections caused by *Es. coli* and *Staphylococcus* species due to their clinical importance. For this study, we selected five different *Es. coli* strains (ATCC 25922, ATCC 4157, ATCC 35218, ATCC 12435, and ATCC 10798). One of these five strains is a β-lactam-resistant *Es. coli* (ATCC 35218). We have included a *Sta. aureus* strain (ATCC 12600) as another example of drug-resistant bacteria (resistant to Cloxacillin) in our study (Marcos et al., 1999). The MIC values for these bacterial strains are provided in Figure 3. The data from this study indicate that all aminoglycosides are very potent against three of the *Es. coli* strains (ATCC 4157, ATCC 12435, and ATCC 10798), with an MIC of 0.2–7.2 μM. Ribostamycin was less effective against two of the *Es. coli* strains (ATCC 25922 and ATCC 35218), with an MIC value of 29.0 and 57.9 μM, respectively. We also observed that both *Sta. aureus* strains from this study were not sensitive to ribostamycin (Figure 3, MIC of 57.9 and 115.8 μM). These data suggest that inherent antibiotic resistance to a β-lactam antibiotic makes the bacterial strain less sensitive to ribostamycin. As a comparison, it has been reported that methicillin-resistant *Sta. aureus* (MRSA) isolates show resistance to gentamicin (MIC = 134–536 mM) and amikacin (MIC = 55–437 mM; Freitas et al., 1999). Despite the low potency against these bacterial strains, ribostamycin is highly effective against majority of the *Es. coli* and a *Sta. epidermidis* strains (Figure 3).

### Antibacterial Activity of Ribostamycin in Combination With EDTA

**Figure 4:**
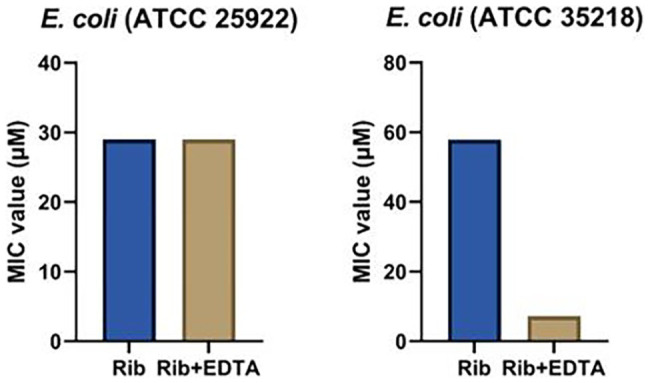
Minimum inhibitory concentration (MIC) values of ribostamycin co-treatment with ethylenediaminetetraacetic acid (EDTA) against *Es. coli* strains.

EDTA, a small molecule metal ion chelator, was explored as potential adjuvant to improve the antibacterial spectrum of tested aminoglycosides (Brown and Richards, 1965; Leive, 1965; Sparks et al., 1994; Lambert et al., 2004; Martin-Visscher et al., 2011; Lebeaux et al., 2014). The first step in this co-treatment approach is to determine a suitable EDTA concentration that is non-toxic to bacteria used in our study. We screened a series of concentrations of EDTA from 0.1 to 52.6 mM, to determine a non-toxic EDTA concentration. We found that the MIC values of EDTA against *Es. coli* was 6,600 mM (2.5 mg/ml), *Sta. aureus* was 0.4 mM (0.16 mg/ml), and *P. aeruginosa* was 13,200 mM (5 mg/ml). To ensure that a low EDTA concentration was used for our studies, we settled with 2,640 μM concentration of EDTA for the combination treatment. When treated with ribostamycin and EDTA (2,640 μM), the MIC value of ribostamycin against first *Es. coli* strain (ATCC 25922) was unaffected and remained as 29.0 μM (Table 1 and Figure 4). However, for the second *Es. coli* strain (ATCC 35218), the MIC value was enhanced from 57.9 to 7.2 μM (Figure 4), which was a remarkable 8-fold improvement in potency. Based on this, we strongly believe that a combination approach using EDTA is a useful strategy to sensitize *Es. coli* strains toward ribostamycin. We also tested the ability of EDTA to sensitize *Sta. aureus* and *P. aeruginosa* (Table 1). EDTA was found to be toxic to *Sta. aureus* at very low concentration (MIC = 0.4 mM); hence, it limited our ability to improve the MIC of ribostamycin against *Sta. aureus*. On the other hand, *P. aeruginosa* did not respond to EDTA-based combination treatment under the tested conditions. It appears, that for different types of bacteria, EDTA concentration needs to be optimized separately, based on the MIC value of EDTA against each organism. Based on the mechanism of action of aminoglycosides, it is known that aminoglycoside class antibiotics exhibit bactericidal activity. Hence, it is reasonable to claim that ribostamycin alone or in combination with EDTA is causing bactericidal activity. Furthermore, there is sufficient literature precedent to support that EDTA acts synergistically with aminoglycosides to improve the inherent antibacterial activity against bacterial pathogens (Lebeaux et al., 2015).

**Table 1:** Ribostamycin co-treatment with EDTA against Gram-positive and Gram-negative bacteria.

	MIC value (μM)		
	Ribostamycin	EDTA*^a^*	Ribostamycin + EDTA (2,640 μM)
*Es. coli* (ATCC 35218)	57.9	6,600	7.2
*Es. coli* (ATCC 25922)	29.0	6,600	29.0
*Sta. aureus* (ATCC 12600)	29.0	0.4	<0.1
*P. aeruginosa* (ATCC 27853)	>116	13,200	>116

EDTA single treatment concentrations tested against *Es. coli* and *Sta. aureus*: 52.6, 26.3, 13.2, 6.6, 3.3, 1.6, 0.8, 0.4, 0.2, and 0.1 mM.

a MIC of EDTA was determined to be the lowest concentration inhibited bacterial growth by itself.

### *In vitro* Cytotoxicity of Aminoglycosides

We also determined the nephrotoxicity of ribostamycin and netilmicin in two kidney cell lines, HEK293 and MDCK cells. For a comparison, we also tested the nephrotoxicity of gentamicin, isepamicin, and amikacin (Table 2). The IC_50_ values of all the antibiotics were well above 100 mM in HEK293 cells. The IC_50_ values of gentamicin and netilmicin were about 32 mM while other antibiotics were well above 100 mM in MDCK cells. Based on the data, it is evident that ribostamycin, the aminoglycoside of interest to us, shows no toxicity against the kidney cell lines. Moreover, ribostamycin behaves similar to other aminoglycosides in terms of cytotoxicity.

**Table 2:** Cytotoxicity of aminoglycosides against two different kidney cell lines.

	IC_50_ value ± SD*^a^* (mM)	
	HEK293	MDCK
Gentamicin sulfate	>100	32.5 ± 0.8
Isepamicin sulfate	>100	>100
Netilmicin	>100	32.8 ± 2.5
Amikacin	>100	>100
Ribostamycin	>100	>100

a IC_50_ values are represented as mean ± SD of at least three independent experiments performed in triplicate.

## Discussion

Antibiotic resistance in Gram-negative bacteria, including *Es. coli* and *P. aeruginosa* strains, is a growing health problem in human and veterinary health. Aminoglycosides have been identified as potent natural antibiotics; yet, there has been limited data on the antibiotic profile and kidney toxicity of some members within aminoglycosides. More specifically, ribostamycin has not been extensively studied against bacterial pathogens. Our study investigates the spectrum of activity of ribostamycin and other members of aminoglycoside family. Our initial screening indicated that all tested aminoglycosides (gentamicin, amikacin, netilmicin, isepamicin, and ribostamycin) are highly potent against *H. influenzae*, *Sta. epidermidis*, and *Str. pneumoniae*. These strains have been more commonly implicated with lung infections (Jones, 2010). The MIC values we have determined for the common aminoglycosides, including gentamicin, netilmicin, and amikacin, are in agreement with the standard MIC values reported in the literature (Chaudhary et al., 2012; Lebeaux et al., 2015). We are particularly excited about our data showing that netilmicin and ribostamycin, two of the less commonly studied aminoglycosides, showed an MIC value as low as 0.1 μM. It is important to note that ribostamycin showed comparable activity against these three strains of bacteria as gentamicin. It is also important to note that netilmicin is more active against *P. aeruginosa* and *En. faecalis* compared to gentamicin. One limitation of ribostamycin is its inability to kill *P. aeruginosa* and *En. faecalis*. However, this observation is a key in, perhaps, discovering new derivatives of ribostamycin to target these resistant bacteria. Next phase of our investigation focused on evaluating these aminoglycosides against a panel of bacteria that cause GI infections. GI infections caused *via* foodborne bacterial pathogens, such as *Es. coli*, have been a major clinical problem. The CDC reports that *Es. coli* infections account for approximately 265,000 infections each year in the US. Such infections are frequently linked to contaminated ground meat and vegetables, such as romaine lettuce (CDC, 2020). From our initial screening, we found that ribostamycin showed promising antibacterial activity against several bacteria, including five *Es. coli* strains (MIC of 0.9–29.0 μM). Ribostamycin is less effective against a β-lactam resistant *Es. coli* and *Sta. aureus* strains. Although this is a limitation, the observation gave us a reason to investigate alternative approaches to improve the efficacy of ribostamycin against these less sensitive pathogens. We also noticed that netilmicin is highly potent against all eight pathogens tested, with MIC values at least two times better than that of gentamicin. For all seven different bacteria tested, the MIC value of netilmicin is less than 5.6 μM, indicating netilmicin being a promising lead for further biological studies.

Since ribostamycin exhibited low activity against two *Es. coli* strains and two *Sta. aureus* strains, we investigate an adjuvant-based strategy to improve the efficacy of ribostamycin against these bacteria. One of the factors that affect the efficacy of aminoglycosides relates to their inability to get into the bacterial cell *via* passive diffusion, due to their highly polar nature. Traditionally, aminoglycosides enter bacterial cell *via* active transport and rely on the proton motive force (Jana and Deb, 2006). For this reason, anaerobic bacteria are generally resistant to aminoglycosides. Small molecule induced aminoglycoside uptake has been a useful strategy to overcome drug resistance relating to poor drug transport into bacterial cells (Lebeaux et al., 2014; Radlinski et al., 2019). EDTA, a small molecule metal ion chelator, has been reported as a highly effective additive that increases bacterial membrane permeability and improves the efficacy of antibiotics (Brown and Richards, 1965; Leive, 1965; Sparks et al., 1994; Lambert et al., 2004; Martin-Visscher et al., 2011; Lebeaux et al., 2014). EDTA has been effectively used in combination with many antibiotics, including gentamicin and amikacin, where a synergistic activity with these antibiotics has been observed (Lambert et al., 2004; Lebeaux et al., 2015; Umerska et al., 2018; Khazandi et al., 2019). Based on literature precedent, we hypothesized that the efficacy of ribostamycin against resistant bacteria can be improved *via* a combination treatment with an adjuvant, such as EDTA.

We found that EDTA exhibited no toxicity against the β-lactam-resistant *Es. coli* (ATCC 35218) up to 6.6 mM concentration. When this strain of *Es. coli* was treated with ribostamycin, in combination with EDTA (2.6 mM), the MIC of ribostamycin was improved considerably to 7.2 μM. This remarkable increase shows that EDTA can be an effective adjuvant to overcome ribostamycin resistance in some strains of *Es. coli*. This antibacterial activity improvement is achieved with a 2.5-fold lower EDTA concentration than the measured MIC value of EDTA against *Es. coli*. It appears that EDTA at the tested concentration (2.6 mM) is very effective in destabilizing the *Es. coli* membrane and allowing the uptake of ribostamycin into the cell. The same strategy did not work against *Sta. aureus* and *P. aeruginosa*. We suspect that the EDTA concentration needs to be adjusted to each type of pathogen, as their sensitivity seems to be different. In the case of *Sta. aureus*, EDTA is too toxic, as Gram-positive bacteria lack the outer membrane. In the case of *P. aeruginosa*, a much higher concentration of EDTA is likely needed, perhaps, due to their inherent low outer membrane permeability and presence of efflux pumps (Pang et al., 2019). Yet, our findings suggest that ribostamycin can be made effective in eradicating drug-resistant *Es. coli*. Due to its mechanism of action and literature reports (Lebeaux et al., 2015), it is likely that EDTA acts in a synergistic fashion to improve the antibacterial activity of ribostamycin.

EDTA is a highly polar molecule due to the presence of two amino groups and four carboxylic acid motifs, making it less permeable through cell membrane (Drisko, 2018). As our goal is to treat GI infections, where the antibiotic (ribostamycin) and the adjuvant (EDTA) are expected to remain in the GI tract, we anticipate that there is minimal concern regarding systemic toxicity of EDTA. Moreover, EDTA is approved as a chelating agent for the treatment of heavy metal poisoning by the US Food and Drug Administration (US FDA; Wax, 2013) and widely used as component of pharmaceutical formulations. Hence, the combination treatment approach we report here is a highly viable approach to improve the efficacy of ribostamycin against GI infection causing pathogens.

One of the major clinical concerns of aminoglycosides is their nephrotoxicity (Tulkens, 1999). Due to limited toxicity data available for the netilmicin and ribostamycin, which are of high interest to us, it is important to evaluate and disclose their toxicity against kidney cell lines. Based on the results, the IC_50_ values for all aminoglycosides are more than 100 mM against HEK293 cell line. The cytotoxicity for ribostamycin is more than 100 mM against MDCK cell line as well (Table 2). Therefore, it is a clear evident that ribostamycin and netilmicin exhibit no toxicity against the kidney cells. During the application of ribostamycin for the treatment of GI infection, we anticipate minimal absorption from the GI tract. Yet, even if some amount of ribostamycin gets absorbed, our cytotoxicity data indicate that ribostamycin and netilmicin are less likely to cause adverse nephrotoxicity. During the combination treatment of ribostamycin or any other antibiotics with EDTA, the toxicity profile against the kidney cell lines is a valid concern. It has been validated that EDTA has an IC_50_ value of 3.4 mM against HEK293 and MDCK cell lines (Khazandi et al., 2019). During our evaluation, we used 2.6 mM EDTA to effectively improve the antibacterial activity of ribostamycin, which is considerably lower than the reported IC_50_ value of EDTA against the two kidney cell lines. Hence, we anticipate no toxicity concern against kidney cell lines for the combination treatment at the tested concentration. Moreover, EDTA is clinically used for chelation therapy as per FDA guidelines (Wax, 2013).

In conclusion, we have evaluated the antibacterial activity of five different aminoglycosides, including netilmicin and ribostamycin, which are classified as “restricted level” and “special level” antibiotics, respectively, in China. Both netilmicin and ribostamycin exhibit very potent activity against several pathogenic bacteria at MIC of 0.2–7.2 μM. Our evaluation of ribostamycin against several GI infection causing bacteria shows that ribostamycin is potently against many different *Es. coli* strains. We also identified EDTA as a small molecule adjuvant to enhance the efficacy of ribostamycin against a drug-resistant *Es. coli* strain, where an 8-fold improvement of the MIC value of ribostamycin is achieved. Finally, our nephrotoxicity study shows that ribostamycin is essentially non-toxic toward two different kidney cell lines, with an IC_50_ of greater than 100 mM. Based on our biological evaluation, ribostamycin shows great promise as a useful therapeutic option to eradicate the pathogens that cause GI infections by itself or in combination with EDTA.

## Data Availability Statement

The raw data supporting the conclusions of this article will be made available by the authors, without undue reservation.

## Author Contributions

SY and Z-SC conceived and designed the experiment. JK, Z-XW, and LW performed the experiments. SY, Z-SC, JK, Z-XW, and LW analyzed the data and wrote the paper. All authors contributed to the article and approved the submitted version.

### Conflict of Interest

The authors declare that the research was conducted in the absence of any commercial or financial relationships that could be construed as a potential conflict of interest.

## References

[ref1] Avent M. L. Rogers B. A. Cheng A. C. Paterson D. L. (2011). Current use of aminoglycosides: indications, pharmacokinetics and monitoring for toxicity. Intern. Med. J. 41, 441–449. 10.1111/j.1445-5994.2011.02452.x, PMID: 21309997

[ref2] Baraboutis I. G. Tsagalou E. P. Lepinski J. L. Papakonstantinou I. Papastamopoulos V. Skoutelis A. T.. (2010). Primary *Staphylococcus aureus* urinary tract infection: the role of undetected hematogenous seeding of the urinary tract. Eur. J. Clin. Microbiol. Infect. Dis. 29, 1095–1101. 10.1007/s10096-010-0967-2, PMID: 20703891

[ref3] Beauchamp D. Labrecque G. (2001). Aminoglycoside nephrotoxicity: do time and frequency of administration matter? Curr. Opin. Crit. Care 7, 401–408. 10.1097/00075198-200112000-00006, PMID: 11805542

[ref4] Beceiro A. Tomás M. Bou G. (2013). Antimicrobial resistance and virulence: a successful or deleterious association in the bacterial world? Clin. Microbiol. Rev. 26, 185–230. 10.1128/CMR.00059-12, PMID: 23554414 PMC3623377

[ref5] Brown M. R. W. Richards R. M. E. (1965). Effect of ethylenediamine tetraacetate on the resistance of *Pseudomonas aeruginosa* to antibacterial agents. Nature 207, 1391–1393. 10.1038/2071391a0, PMID: 4287358

[ref6] Carmichael J. DeGraff W. G. Gazdar A. F. Minna J. D. Mitchell J. B. (1987). Evaluation of a tetrazolium-based semiautomated colorimetric assay: assessment of chemosensitivity testing. Cancer Res. 47, 936–942. PMID: 3802100

[ref45] CDC (2019). Antibiotic resistance threats in the United States. Atlanta, GA, USA: Department of Health and Human Services, CDC.

[ref7] CDC (2020). Reports of selected *Es. coli* outbreak inverstigations. Available at: https://www.cdc.gov/ecoli/outbreaks.html (Accessed March 9, 2020).

[ref8] Chaudhary M. Naidu G. K. Kumar S. Payasi A. (2012). Comparative antibacterial activity of novel semisynthetic antibiotic: etimicin sulfphate and other aminoglycosides. World J. Microbiol. Biotechnol. 28, 3365–3371. 10.1007/s11274-012-1148-5, PMID: 22983905

[ref9] Clinical and Laboratory Standards Institute (2012). Methods for dilution antimicrobial susceptibility tests for bacteria that grow aerobically: Approved standard—9th Edn. Vol. 32 Wayne, PA, USA: CLSI. ISBN: 1-56238-783-9.

[ref10] Drisko J. A. (2018). “Chapter 107: chelation therapy” in Integrative medicine. 4th *Edn* ed. Rakel D. (Philadelphia, PA: Elsevier), 1004.e3–1015.e3.

[ref11] Durante-Mangoni E. Grammatikos A. Utili R. Falagas M. E. (2009). Do we still need the aminoglycosides? Int. J. Antimicrob. Agents 33, 201–205. 10.1016/j.ijantimicag.2008.09.001, PMID: 18976888

[ref12] EUCAST (2003). EUCAST discussion document E.Dis 5.1 March 2003: determination of minimum inhibitory concentrations (MICs) of antibacterial agents by broth dilution. Clin. Microbiol. Infect. 9, 9–15. 10.1046/j.1469-0691.2003.00790.x 11168187

[ref13] EUCAST of ESCMID (2003). EUCAST definitive document E.DEF 3.1, June 2000: determination of minimum inhibitory concentrations (MICs) of antibacterial agents by agar dilution. Clin. Microbiol. Infect. 9, 509–515. 10.1046/j.1469-0691.2000.00142.x, PMID: 11168187

[ref14] Falagas M. E. Karageorgopoulos D. E. Georgantzi G. G. Sun C. Wang R. Rafailidis P. I. (2012). Susceptibility of Gram-negative bacteria to isepamicin: a systematic review. Expert Rev. Anti-Infect. Ther. 10, 207–218. 10.1586/eri.11.170, PMID: 22339194

[ref15] Freitas F. I. Guedes-Stehling E. Siqueira-Junior J. P. (1999). Resistance to gentamicin and related aminoglycosides in *Staphylococcus aureus* isolated in Brazil. Lett. Appl. Microbiol. 29, 197–201. 10.1046/j.1365-2672.1999.00617.x, PMID: 10530041

[ref16] Frieri M. Kumar K. Boutin A. (2017). Antibiotic resistance. J. Infect. Public Health 10, 369–378. 10.1016/j.jiph.2016.08.007, PMID: 27616769

[ref17] Garneau-Tsodikova S. Labby K. J. (2016). Mechanism of resistance to aminoglycoside antibiotics: overview and perspectives. Medchemcomm 7, 11–27. 10.1039/C5MD00344J, PMID: 26877861 PMC4752126

[ref18] Hancock R. E. W. (1981). Aminoglycoside uptake and mode of action-with special reference to streptomycin and gentamicin. II. Effects of aminoglycosides on cells. J. Antimicrob. Chemother. 8, 429–445. 10.1093/jac/8.6.429, PMID: 7037727

[ref19] Hillerton J. E. Berry E. A. (2005). Treating mastitis in the cow—a tradition or an archaism. J. Appl. Microbiol. 98, 1250–1255. 10.1111/j.1365-2672.2005.02649.x, PMID: 15916638

[ref20] Horibe T. Nagai H. Sakakibara K. Hagiwara Y. Kikuchi M. (2001). Ribostamycin inhibits the chaperone activity of protein disulfide isomerase. Biochem. Biophys. Res. Commun. 289, 967–972. 10.1006/bbrc.2001.6105, PMID: 11741285

[ref21] Houghton J. L. Green K. D. Chen W. Garneau-Tsodikova S. (2010). The future of aminoglycosides: the end or renaissance? Chembiochem 11, 880–902. 10.1002/cbic.200900779, PMID: 20397253

[ref22] Jana S. Deb J. K. (2006). Molecular understanding of aminoglycoside action and resistance. Appl. Microbiol. Biotechnol. 70, 140–150. 10.1007/s00253-005-0279-0, PMID: 16391922

[ref23] Jones R. N. (2010). Microbial etiologies of hospital-acquired bacterial pneumonia and ventilator-associated bacterial pneumonia. Clin. Infect. Dis. 51, S81–S87. 10.1086/653053, PMID: 20597676

[ref24] Kapoor G. Saigal S. Elongavan A. (2017). Action and resistance mechanisms of antibiotics: a guide for clinicians basic anatomy of bacterial cell. J. Anaesthesiol. Clin. Pharmacol. 33, 300–305. 10.4103/joacp.JOACP_349_15, PMID: 29109626 PMC5672523

[ref25] Khazandi M. Pi H. Chan W. Y. Ogunniyi A. D. Sim J. X. F. Venter H.. (2019). In vitro antimicrobial activity of robenidine, ethylenediaminetetraacetic acid and polymyxin B nonapeptide against important human and veterinary pathogens. Front. Microbiol. 10:837. 10.3389/fmicb.2019.00837, PMID: 31105656 PMC6494957

[ref26] Kotra L. P. Haddad J. Mobashery S. (2000). Aminoglycosides: perspectives on mechanisms of action and resistance and strategies to counter resistance. Antimicrob. Agents Chemother. 44, 3249–3256. 10.1128/AAC.44.12.3249-3256.2000, PMID: 11083623 PMC90188

[ref27] Krause K. M. Serio A. W. Kane T. R. Connolly L. E. (2016). Aminoglycosides: an overview. Cold Spring Harb. Perspect. Med. 6:a027029. 10.1101/cshperspect.a027029, PMID: 27252397 PMC4888811

[ref28] Lambert R. J. W. Hanlon G. W. Denyer S. P. (2004). The synergistic effect of EDTA/antimicrobial combinations on *Pseudomonas aeruginosa*. J. Appl. Microbiol. 96, 244–253. 10.1046/j.1365-2672.2004.02135.x, PMID: 14723685

[ref29] Lebeaux D. Ghigo J. -M. Beloin C. (2014). Biofilm-related infections: bridging the gap between clinical management and fundamental aspects of recalcitrance toward antibiotics. Microbiol. Mol. Biol. Rev. 78, 510–543. 10.1128/MMBR.00013-14, PMID: 25184564 PMC4187679

[ref30] Lebeaux D. Leflon-Guibout V. Ghigo J. M. Beloin C. (2015). In vitro activity of gentamicin, vancomycin or amikacin combined with EDTA or L-arginine as lock therapy against a wide spectrum of biofilm-forming clinical strains isolated from catheter-related infections. J. Antimicrob. Chemother. 70, 1704–1712. 10.1093/jac/dkv044, PMID: 25712314

[ref31] Leive L. (1965). A nonspecific increase in permeability in *Escherichia coli* produced by Edta. Proc. Natl. Acad. Sci. U. S. A. 53, 745–750. 10.1073/pnas.53.4.745 14324530 PMC221061

[ref32] Lopez-Novoa J. M. Quiros Y. Vicente L. Morales A. I. Lopez-Hernandez F. J. (2011). New insights into the mechanism of aminoglycoside nephrotoxicity: an integrative point of view. Kidney Int. 79, 33–45. 10.1038/ki.2010.337, PMID: 20861826

[ref33] Magnet S. Blanchard J. S. (2005). Molecular insights into aminoglycoside action and resistance. Chem. Rev. 105, 477–497. 10.1021/cr0301088, PMID: 15700953

[ref34] Marcos J. Y. Soriano A. C. Salazar M. S. Moral C. H. Ramos S. S. Smeltzer M. S.. (1999). Rapid identification and typing of *Staphylococcus aureus* by PCR-restriction fragment length polymorphism analysis of the aroA gene. J. Clin. Microbiol. 37, 570–574. 10.1128/JCM.37.3.570-574.1999, PMID: 9986814 PMC84472

[ref35] Martin-Visscher L. A. Yoganathan S. Sit C. S. Lohans C. T. Vederas J. C. (2011). The activity of bacteriocins from *Carnobacterium maltaromaticum* UAL307 against Gram-negative bacteria in combination with EDTA treatment. FEMS Microbiol. Lett. 317, 152–159. 10.1111/j.1574-6968.2011.02223.x, PMID: 21255070

[ref36] Masi M. Réfregiers M. Pos K. M. Pagès J. M. (2017). Mechanisms of envelope permeability and antibiotic influx and efflux in Gram-negative bacteria. Nat. Microbiol. 2:17001. 10.1038/nmicrobiol.2017.1, PMID: 28224989

[ref37] Miller G. H. Arcieri G. Weinstein M. J. Waitz J. A. Al M. E. T. (1976). Biological activity of netilmicin, a broad-spectrum sei, pisynthetic aminoglycoside antibiotic. Antimicrob. Agents Chemother. 10, 827–836. 10.1128/AAC.10.5.827, PMID: 1008541 PMC429844

[ref38] Muder R. R. Brennen C. Rihs J. D. Wagener M. M. Obman A. Stout J. E.. (2006). Isolation of *Staphylococcus aureus* from the urinary tract: association of isolation with symptomatic urinary tract infections and subsequent staphylococcal bacteremia. Clin. Infect. Dis. 42, 46–50. 10.1086/498518, PMID: 16323090

[ref39] Nakashima T. Teranishi M. Hibi T. Kobayashi M. Umemura M. (2000). Vestibular and cochlear toxicity of aminoglycosides—a review. Acta Otolaryngol. 120, 904–911. 10.1080/00016480050218627, PMID: 11200584

[ref40] Nikaido H. Nakae T. (1980). The outer membrane of Gram-negative bacteria. Adv. Microb. Physiol. 20, 163–250. 10.1016/S0065-2911(08)60208-8 394591

[ref41] Pang Z. Raudonis R. Glick B. R. Lin J. T. Cheng Z. (2019). Antibiotic resistance in *Pseudomonas aeruginosa*: mechanisms and alternative therapeutic strategies. Biotechnol. Adv. 37, 177–192. 10.1016/j.biotechadv.2018.11.013, PMID: 30500353

[ref42] Parks T. Hill A. V. Chapman S. J. (2012). The perpetual challenge of infectious diseases. N. Engl. J. Med. 367:90. 10.1056/NEJMc1204960, PMID: 22762339

[ref43] Radlinski L. C. Rowe S. E. Brzozowski R. Wilkinson A. D. Huang R. Eswara P.. (2019). Chemical induction of aminoglycoside uptake overcomes antibiotic tolerance and resistance in *Staphylococcus aureus*. Cell Chem. Biol. 26, 1355.e4–1364.e4. 10.1016/j.chembiol.2019.07.009, PMID: 31402316 PMC6800641

[ref44] Ramirez M. S. Tolmasky M. E. (2017). Amikacin: uses, resistance, and prospects for inhibition. Molecules 22, 2267–2270. 10.3390/molecules22122267, PMID: 29257114 PMC5889950

[ref46] Rizzi M. D. Hirose K. (2007). Aminoglycoside ototoxicity. Curr. Opin. Otolaryngol. Head Neck Surg. 15, 352–357. 10.1097/MOO.0b013e3282ef772d, PMID: 17823553

[ref47] Schabauer A. Zutz C. Lung B. Wagner M. Rychli K. (2018). Gentisaldehyde and its derivative 2,3-dihydroxybenzaldehyde show antimicrobial activities against bovine mastitis *Staphylococcus aureus*. Front. Vet. Sci. 5:148. 10.3389/fvets.2018.00148, PMID: 30050910 PMC6050399

[ref48] Selimoglu E. (2007). Aminoglycoside-induced ototoxicity. Curr. Pharm. Des. 13, 119–126. 10.2174/138161207779313731, PMID: 17266591

[ref49] Shankar N. Lockatell C. V. Baghdayan A. S. Drachenberg C. Gilmore M. S. Johnson D. E. (2001). Role of *Enterococcus faecalis* surface protein Esp in the pathogenesis of ascending urinary tract infection. Infect. Immun. 69, 4366–4372. 10.1128/IAI.69.7.4366-4372.2001, PMID: 11401975 PMC98508

[ref50] Sparks T. A. Kemp D. T. Wooley R. E. Gibbs P. S. (1994). Antimicrobial effect of combinations of EDTA-Tris and amikacin or neomycin on the microorganisms associated with otitis externa in dogs. Vet. Res. Commun. 18, 241–249. 10.1007/BF01839190, PMID: 7831753

[ref51] Steinbuch K. B. Fridman M. (2016). Mechanisms of resistance to membrane-disrupting antibiotics in Gram-positive and Gram-negative bacteria. Medchemcomm 7, 86–102. 10.1039/C5MD00389J

[ref52] Stryjewski M. E. Corey G. R. (2014). Methicillin-resistant *Staphylococcus aureus*: an evolving pathogen. Clin. Infect. Dis. 58, S10–S19. 10.1093/cid/cit613, PMID: 24343827

[ref53] Tulkens P. M. (1999). Aminoglycosides: nephrotoxicity. Antimicrob. Agents Chemother. 43, 1003–1012. 10223907 10.1128/aac.43.5.1003 PMC89104

[ref54] Umerska A. Strandh M. Cassisa V. Matougui N. Eveillard M. Saulnier P. (2018). Synergistic effect of combinations containing EDTA and the antimicrobial peptide AA230, an arenicin-3 derivative, on Gram-negative bacteria. Biomolecules 8:122. 10.3390/biom8040122, PMID: 30360557 PMC6315934

[ref55] Vaara M. (1992). Agents that increase the permeability of the outer membrane. Microbiol. Rev. 56, 395–411. 10.1128/MMBR.56.3.395-411.1992, PMID: 1406489 PMC372877

[ref56] Vong K. Auclair K. (2012). Understanding and overcoming aminoglycoside resistance caused by N-6ʹ-acetyltransferase. Medchemcomm 3, 397–407. 10.1039/c2md00253a, PMID: 28018574 PMC5179255

[ref57] Wax P. M. (2013). Current use of chelation in American health care. J. Med. Toxicol. 9, 303–307. 10.1007/s13181-013-0347-2, PMID: 24113860 PMC3846961

[ref58] Woodford N. Turton J. F. Livermore D. M. (2011). Multiresistant Gram-negative bacteria: the role of high-risk clones in the dissemination of antibiotic resistance. FEMS Microbiol. Rev. 35, 736–755. 10.1111/j.1574-6976.2011.00268.x, PMID: 21303394

[ref59] Wright H. Bonomo R. A. Paterson D. L. (2017). New agents for the treatment of infections with Gram-negative bacteria: restoring the miracle or false dawn? Clin. Microbiol. Infect. 23, 704–712. 10.1016/j.cmi.2017.09.001, PMID: 28893690

[ref60] Xu A. Zheng B. Xu Y. C. Huang Z. G. Zhong N. S. Zhuo C. (2016). National epidemiology of carbapenem-resistant and extensively drug-resistant Gram-negative bacteria isolated from blood samples in China in 2013. Clin. Microbiol. Infect. 22, S1–S8. 10.1016/j.cmi.2015.09.015, PMID: 26846351

[ref61] Yoganathan S. Miller S. J. (2015). Sturcture diversification of vancomycin through peptide-catalyzed, site-selective lipidation: a catalysis-based approach to combat glycopeptide-resistant pathogens. J. Med. Chem. 58, 2367–2377. 10.1021/jm501872s, PMID: 25671771 PMC4364393

[ref62] Yoganathan S. Sit C. S. Vederas J. C. (2011). Chemical synthesis and biological evaluation of gallidermin-siderophore conjugates. Org. Biomol. Chem. 9, 2133–2141. 10.1039/c0ob00846j, PMID: 21290068

[ref63] Yoganathan S. Yin N. He Y. Mesleh M. F. Gu Y. G. Miller S. J. (2013). An efficient chemical synthesis of carboxylate-isostere analogs of daptomycin. Org. Biomol. Chem. 11, 4680–4685. 10.1039/c3ob40924d, PMID: 23752953 PMC4033608

