# Supplementary material for: Exploration of Antibiotic Activity of Aminoglycosides, in Particular Ribostamycin Alone and in Combination With Ethylenediaminetetraacetic Acid Against Pathogenic Bacteria
Source: Front Microbiol. 2020 Jul 29;11:1718. doi: 10.3389/fmicb.2020.01718 (PMC7403490; doi:10.3389/fmicb.2020.01718)
Supplement: Supplementary file 1 [file Data_Sheet_1.PDF]

*Supporting Information*

# Exploration of antibiotic activity of ribostamycin alone and in combination with EDTA against pathogenic bacteria

Jing Kong[1], Zhuoxun Wu[1], Liuya Wei [1, 2], Zhe-Sheng Chen[1*] and Sabesan Yoganathan[1*]

[1]Department of Pharmaceutical Sciences, College of Pharmacy and Health Sciences, St. John's University, Queens, New York, 11439, USA

[2]School of Pharmacy, Weifang Medical University, Weifang, 261053, China

***Correspondence:** Corresponding Authors' e-mail: yoganats@stjohns.edu (Dr. Yoganathan); chenz@stjohns.edu (Dr. Chen)

## 1. Experimental procedures for biological evaluations:

**MIC determination for combination with EDTA:** Ribostamycin solution (100 µL) with different concentrations (64, 32, 16, 8, 4, 2, 1, 0.5 to 0.25 µg/mL) was added to each well. Then, 5 µL of EDTA (1 mg/mL) solution was added to each well. Subsequently, 100 µL of ~$5x10^5$ CFU/mL inoculum was added into each well. The plates were then incubated at 37 °C for 14 hours. The $OD_{600}$ was recorded using a microplate reader. MICs are reported as the lowest concentration at which no bacterial growth was observed.

**MIC determination using resazurin dye:** Sterile resazurin solution (0.02% by weight in water) was added into the 96-well plate after 16 hours of incubation in a plate reader. The plate was further incubated for another 3 hours to let the bacteria react with the resazurin dye at 37 °C. After incubation, color change was used to estimate the MIC values. The well color changed from blue to purple/pink was estimated to be the MIC value.

**Cytotoxicity assay:** HEK293 and MDCKII cells were selected to perform the nephrotoxicity assay. Nephrotoxicity of the antibiotics was determined by the MTT assay (Carmichael *et al.* 1987). Briefly, cells were collected and seeded evenly into 96-well plates ($5 \times 10^3$ cells per well) and were maintained overnight. At the next day, different concentrations of each antibiotics were added into the designated wells. After 68 h of incubation, 20 µL MTT solution (4 mg/mL) was added to each well and the cells were further incubated for additional 4 h in the incubator. Then the supernatant was discarded and 100 µL of DMSO was added to dissolve the formazan crystals. The light absorbance was determined by using an AccuSkan$^{TM}$ GO UV/Vis Microplate Spectrophotometer (Fisher Sci., Fair Lawn, NJ) at a wavelength of 570 nm.

## 2. MIC values for the aminoglycosides tested

Table 1. Antibacterial activity of aminoglycosides against select Gram-positive and Gram-negative bacteria

| | MIC value (μM) | | | | |
|---|---|---|---|---|---|
| | Gentamicin | Amikacin | Netilmicin | Isepamicin | Ribostamycin |
| *E. coli* (ATCC 25922) | 13.9 | 27.3 | 5.6 | 24.0 | 29.0 |
| *P. aeruginosa* (ATCC 27853) | 3.5 | 3.4 | 2.8 | 6.0 | >115.8 |
| *H. influenzae* (ATCC 49247) | 0.9 | 3.4 | 0.1 | 3.0 | 0.5 |
| *S. epidermidis* (ATCC 12228) | 0.4 | 3.4 | 0.2 | 6.0 | 3.6 |
| *S. aureus* (ATCC 29213) | 3.5 | 13.7 | 5.6 | 6.0 | 57.9 |
| *E. faecalis* (ATCC 29212) | 27.8 | >109.3 | 5.6 | 95.9 | >115.8 |
| *S. pneumoniae* (ATCC 49619) | 6.9 | 13.7 | 0.1 | 6.0 | 7.2 |

Table 2. Antibacterial activity of aminoglycosides against select GI infection causing pathogens

| | MIC value (μM) | | | | |
|---|---|---|---|---|---|
| | Gentamicin | Amikacin | Netilmicin | Isepamicin | Ribostamycin |
| *E. coli* (ATCC 25922) | 13.9 | 27.3 | 5.6 | 24.0 | 29.0 |
| *E. coli* (ATCC 4157) | 6.9 | 6.8 | 5.6 | 3.0 | 7.2 |
| *E. coli* (ATCC 35218) | 27.8 | 54.6 | 11.1 | 6.0 | 57.9 |
| *E. coli* (ATCC 12435) | 0.9 | 0.2 | 0.3 | 0.2 | 0.9 |
| *E. coli* (ATCC 10798) | 3.5 | 0.4 | 2.8 | 3.7 | 3.6 |
| *S. epidermidis* (ATCC 12228) | 0.4 | 3.4 | 0.2 | 6.0 | 3.6 |
| *S. aureus* (ATCC 29213) | 3.5 | 13.7 | 5.6 | 6.0 | 57.9 |
| *S. aureus* (ATCC 12600) | 6.9 | 54.6 | 2.8 | 47.9 | 115.8 |
